# TMedicus, on Domestic Quackery

**Published:** 1802-05

**Authors:** 


### To Dr. BRADLEY.

SIR,

I Shall take the liberty of troubling you with a few Remarks upon Quackery, a fubject which has already drawn forth fome valuable Obfervations from your Correfpondents, and one that deferves ftill more to be noticed. It is, however, to a different fpecies of Quackery from that noticed in the Medical Journal to which I wifh at prefent to draw your attention, as it appears to me to be of a more ferious nature than the fale of quack medicines, and I apprehend it to be a growing evil.

The circumftance which I allude to, is that of private families *quacking* themfelves, and prefuming to ufe the moft active remedies in the Materia Medica, with as little fcruple or caution as, a few years ago, the good old ladies would have adminiftered a treacle poffet or an infufion of rofemary. Bark, antimony, calomel, and opium, are now become domeftic remedies. It is by no means uncommon to hear a mother obferve, with much felf-complacency, that her child was indifpofed with a cold, or reftlefs from dentition, but that the diforder had yielded to half a dozen drops of laudanum: Or fhe obferves, that fhe has herfelf been affected with pain at her ftomach, or, as the more learned term it, with fpafm, but thirty drops of laudanum had refcued her from the moft imminent danger. To fuch an height has the ufe of this dangerous medicine rifen, that in a fea-port town of great trade in the north of England, it conftitutes at prefent one of the moft fafhionable drams among the middling and lower claffes. A delicate woman who would be fhocked at the idea of taking fpirits, does not fcruple to fwallow occafionally, a dofe of laudanum to drive away the effects of ennui; and it is very generally the practice where children are committed to the care of hired nurfes, to keep them in a conftant doze by means of opiates, qualified indeed as medicines to difpel wind and *nourish* the bowels. Whence have thefe evils originated? I anfwer, from medical men. It is faid of the late excellent Dr. Fothergill, that he regretted having ufed fpirituous tinctures fo frequently in his practice, as he feared, that in many inftances he had infenfibly paved the way to fpirit drinking; but the unfortunate victims to the more deftructive habit of taking opium, have greater reafon to blame thofe who have indifcreetly taught them this practice. I do not mean to reprobate the ufe of opium, one of the greateft bleffings which Heaven has beftowed upon wretched mortals; it is the prefent indifcriminate and improper ufe of it that I condemn; the practice, now too common of trufting this dangerous remedy medy to the difcretion of the patient, or, what is worfe, to that of an officious nurfe.

The firft wifh that a medical man has, is to cure his patient, the next, and equally important, is to acquire his confidence. When pain ceafes, the fufferer has leifure to contemplate the idol whom he fo lately worfhipped; he finds him, perhaps, ill informed, and, like the frogs in the fable, in the cafe of the good king Log, his admiration ceafes, or is changed into contempt. This has not efcaped the notice of the weaker and more cunning part of the profeffion; and to avoid detection, it has introduced amongft them the cuftom, fo juftly ridiculed by dramatic writers, of retailing the fcandal of the day, or of prattling about the contents of their fhops. Others of the profeffion, who might adorn the moft important ftations, (and fuch men are to be found in every branch of it) have committed the fame error, though from an oppofite caufe, from a franknefs of temper and a wifh to avoid every appearance of craft. Treatifes upon domeftic medicine, and other popular works of this kind, have greatly increafed the mifchief, without diffufing one falutary ray of information; for medicine cannot be learned from books, much lefs from a fingle volume which profeffes to cure the long lift of difeafes 'to which mortality is heir.' The latter fource of evil is probably irremediable, for popularity has charms: Men, from various motives, are fond of commencing authors; and happily, the freedom of the prefs, though teeming with mifchief, ftill continues. But let regular practitioners endeavour to obviate thefe ill effects by becoming more guarded in their general mode of conduct; and let them be as cautious in trufting thefe active and important remedies to the difcretion of the vulgar, as they would be of committing a fharp inftrument to an infant or an idiot.

The practice which now too commonly prevails among phyficians, of fending, or even of allowing their prefcriptions to be fent to a druggift, is fraught with much harm; and muft in time oblige apothecaries, in felf-defence, to arm themfelves with doctorial panoply. For, notwithftanding the *charm* of a diploma, patients in general will prefer an old and refpectable apothecary to a young and inexperienced doctor. It would be well alfo if apothecaries, with a view to check thefe alarming innovations, would avoid purchafing their medicines of fuch druggifts as thus attempt to undermine them. A phyfician who acts with propriety will *order* his prefcription to be fent to an apothecary, in order to fupport an ufeful, and I may venture to add, a refpectable branch of the profeffion; it will alfo prevent the apothecary from looking with jealoufy upon the phyfician, and at the fame time enable him to charge more moderately for his medicines. It may not be an improper caution to add, that the prefcription ought to remain with the apothecary, and he ought to be refponfible for its fafety. The patient has no right to the prefcription, except in the cafe of undertaking a journey, and of being liable to relapfes of the complaint. This caution does not arife from felfifhnefs, it is founded on prudence, and medical men have unjuftly fuffered by their too great readinefs to communicate their mode of practice. In numberlefs inftances, the ufual practice of returning the prefcription to the patient has given origin to a new quack medicine. Befides, when patients know the compofition of remedies, they not unfrequently object to fome of the Ingredients, or fancy they will do no good. Mankind are fond of myftery; and it is more congenial with a fick man's mind to expect relief from the occult qualities of a medicine than from its fenfible virtues. Hence, in a great meafure, arifes the fuccefs of many boafted fecret remedies, which inftantly lofe their efficacy when compounded at an apothecary's fhop. This has lately been extremely well pointed out by the ingenious and learned Dr. Haygarth, in his expofure of a Reverend Quack and his metal fkewers.

Let patients have every fatisfaction given them refpecting the nature and probable termination of their complaints; let them alfo be told the *general plan* of treatment; but it is unneceffary to point out the particular remedies to be employed. How very readily *idle people* fall into the mode of treating trifling complaints without regular advice, and how ready they are to deprive a medical man of his juft emoluments, the following inftance will prove; and I have no doubt that many of your readers will recognize fimilar characters within their circle of practice. Mr. Robert Booby was bred to the fea, which he quitted for the enviable life of a country fquire; but having taken more care to load his coffers than to ballaft his mind, he became a prey to ennui. As he had not a capacity for any other employment, he turned his thoughts to medicine. A parlour clofet was converted into an apothecary's fhop, and he began to difpenfe medicines to the poor of the village. When his family was fick, his furgeon, a man of eminence, and of a frank unfufpecting temper, not only anfwered all his queftions, but fhewed him the mode of compounding his medicines, which Mr. Booby, under pretence of faving trouble by fending for them, but in reality to fave expence, occafionally prepared from his clofet. In this manner they proceeded for fome years, until Mr. Booby, at length, made up all the medicines himfelf, or, to fhew his gratitude, fent for them from a druggift. Thus the emolument of the furgeon was confined fined to a trifling fum, barely adequate to pay for his horfe for occafional vifits; in fhort, he faw his error when too late. It may perhaps be a fubject of congratulation, that we have at prefent fewer *old women* in the profeffion than formerly; yet we ought to treat with due reverence thofe old *ladies* who amufe themfelves with culling of fimples for the good of their poor neighbours. I have one in my neighbourhood, who does wonders with her black and green falves (the green and black bafilicon) which fhe difpenfes in mufcle fhells, and fuppofes to be invaluable fecrets. Her ardor for the profeffion can only excite a fmile, for I believe fhe does no harm. This, however, cannot be faid of another clafs, called bone-fetters, who are real pefts to fociety, and whofe ignorance can only be equalled by their daring impudence. Of their rough and improper modes of practice, almoft every county-hofpital can fhew melancholy inftances. If confiftent with national freedom, the interference of the Legiflature appears in no one inftance to be more requifite than in fuppreffing this noxious tribe. Some of your correfpondents may be able to give a fketch, which cannot fail to be amufing, of one of thefe people, lately deceafed, and very generally known in Lancafhire by the name of the Whitworth doctor.*

Medical men, as guardians of the public health, fhould confider it as a duty incumbent upon them to expofe the dangerous errors of thefe people, and to detect the tricks of quacks in general. An excellent example has been placed before them by a lady of high rank, the duchefs of Montrofe, who condefcended lately, in the public papers, to contradict the affertion of an advertifing female in Long Acre, London, who prefumed to place her Grace's name at the head of a moft refpectable lift of witneffes (all probably in like manner forged) to the efficacy of her Noftrum.

I fhall no further trefpafs upon your patience than to obferve, that Patent Medicines are a difgrace to the Legiflature; they impofe upon the vulgar, who imagine that the wifdom of government has thought it proper to reward the merit of the inventor by a public mark of favor; and perfons who fcruple to pay a trifling bill to an apothecary, throw away ten times that fum upon a vile quack medicine. But while we fpeak with contempt of fuch men as Broad-hum and Sillyman, who *abufe* a credulous public with their botanic fyrup and cordial balm, why do we pafs over in filence thofe who apoftatize from amongft

* It was very common for this perfon to give his druggift an order for a ton of Glauber Salts, and *three ftone* weight of Opium.

amongſt ourſelves? Ought they not to have ſome mark of contempt, or, at leaſt, of diſapprobation attached to them? The Chineſe apoſtate Ching-ſi, ſo celebrated of late for his brown and yellow lozenges, vends at an enormous price jalap and calomel, which country practitioners daily uſe, and have uſed before he was born, and yet calls himſelf a regular practitioner. But we find a more dignified delinquent, no leſs than a Fellow of the College, accuſed of a ſimilar offence in a late elegant and intereſting work, entitled, 'Reports on the Diſeaſes in London', by a phyſician, whoſe great erudition, extenſive practical knowledge, and ſtill more, his active benevolence, render him an ornament to the profeſſion.

MEDICUS

P. S. The Editors of the Phyſical Journal formerly promiſed to notice, occaſionally, the compoſition of Quack remedies; a promiſe which, except in one or two inſtances, appears to have been forgotten: For though ſuch Traſh are ſcarcely worth notice, yet ſome good may accrue from their compoſition being expoſed.

